# Sleep-wake and circadian behavior in brain-specific sirtuin 1 knockout mice and effects of nicotinamide mononucleotide supplementation

**DOI:** 10.1038/s41598-026-57888-2

**Published:** 2026-06-23

**Authors:** Noriaki Sakai, Ryohei Yoshimoto, Daisuke Yamaguchi, Seiji Nishino

**Affiliations:** 1 Department of Psychiatry and Behavioral Sciences, Stanford University School of Medicine, Palo Alto, CA 94304 USA; 2 Department of Life Sciences, Graduate School of Arts and Sciences, The University of Tokyo, Tokyo, Japan; 3 Laboratory for Circuit and Behavioral Physiology, RIKEN Brain Science Institute, Wako, Japan; 4 Department of Gerodontology and Home Care Dentistry, School of Dentistry, Aichi Gakuin University, Aichi, Japan

**Keywords:** Sirtuin 1, NMN, Sleep and wake, Circadian rhythm, Brain-specific knockout, Neurology, Neuroscience, Physiology

## Abstract

Sirtuin 1 (SIRT1) is implicated in sleep regulation, but there have been a limited number of sleep studies related to SIRT1. We aim to determine the impacts of the brain-specific loss of SIRT1 on sleep and circadian rhythm. We first characterized circadian periods and sleep phenotypes in baseline and recovery sleep in brain-specific *Sirt1* knockout (BKO) and littermate wild-type (WT) mice. We found that there were no differences in sleep architecture, amount of each vigilance state, and sleep homeostasis between genotypes, suggesting that brain SIRT1 functionally plays minor roles in sleep-wake regulation. However, in accordance with prior work, loss of *Sirt1* caused decreased delta power during non-REM sleep. In addition, there was no difference in intrinsic circadian periods between genotypes. We also provide the first preclinical evidence of whether nicotinamide mononucleotide (NMN) treatment directly improves sleep by administering it acutely (intraperitoneally for 10 days) and chronically (orally for 2 months). However, neither acute nor chronic treatment changed sleep duration and quality compared to the control session in middle-aged WT and BKO mice. Considering our findings and previous studies, the clinically reported improvement in muscle function and energy metabolism may indirectly contribute to the potential benefits of NMN on sleep quality and fatigue in the elderly.

**Supplementary Information:**

The online version contains supplementary material available at https://doi.org/10.1038/s41598-026-57888-2.

## Introduction

Sirtuins (SIRTs) are NAD⁺-dependent protein deacetylases that play essential roles in various physiological processes, including aging, metabolic regulation, and energy homeostasis. Among seven known mammalian sirtuins, SIRT1 is the most extensively studied and is known to regulate gene expression through histone deacetylation and the interaction with multiple transcription factors in multiple tissues such as the liver, skeletal muscle, and brain^1,2^. It also acts as a sensor of various cellular stressors, including oxidative, metabolic, and genotoxic stress. These stressors can regulate SIRT1 activity, influencing cell survival and potentially balancing between cytoprotective and apoptotic pathways. Due to its diverse cellular functions, it has been linked to the development of a wide range of diseases, including diabetes, cancer, and neurodegenerative disorders^3,4^.

Since SIRT1 activity is closely linked to energy balance and stress responses, it is plausible that it can play a role in modulating sleep-wake dynamics. Previous studies have suggested that SIRT1 contributes to the regulation of sleep and circadian rhythms. Regarding circadian regulation, the mechanisms responsible for the circadian clock control have been the subject of contradictory findings^5,6^. However, results from both in vivo and in vitro studies converge to mutual interaction between core clock genes and SIRT1 activity that is mediated by the oscillating levels of NAD + and nicotinamide phosphoribosyltransferase (*Nampt)* that is involved in NAD+ biosynthesis^7,8^. On the other hand, there have been reported only a few SIRT1-related sleep studies using transgenic mice. Although whole-body SIRT1 knockout (KO) in inbred mice is lethal prenatally, sleep phenotypes were first reported in outbred backgrounds that impaired wakefulness and the blunted delta power were accompanied by wake-active neuron dysfunction in outbred KO mice^9^. Satoh et al. also demonstrated that aged brain-specific SIRT1 overexpressing mice showed better physical activity and higher delta power during non-REM (NREM) sleep compared to age-matched control mice^20^. However, the primary mechanisms through which SIRT1 directly regulates sleep and wake behavior remain unclear.

Nicotinamide mononucleotide (NMN), a key NAD⁺ precursor, has emerged as a promising molecule with anti-aging effects, partly by enhancing SIRT1 activity. Clinical and preclinical studies indicate that NMN supplementation can improve metabolic function and mitigate the progression of age-related physiological declines^11–14^. NMN elevates intracellular NAD⁺ levels, thereby restoring energy homeostasis in peripheral tissues such as muscle and liver. Its impact on the nervous system has also attracted attention, with clinical evidence suggesting that NAD⁺ replenishment by NMN may improve sleep quality, drowsiness, and tiredness^15–17^. Nevertheless, whether these benefits stem from direct actions on the central nervous system or are mediated via peripheral tissues remains a topic of debate.

Therefore, the aims of this study are dual: first, to determine the impacts of the brain-specific loss of SIRT1 on sleep and circadian rhythm; and second to determine the effects of NMN on sleep and wake behavior that have not yet been tested in rodents. To address these aims, we performed baseline characterization and evaluated the impact of both acute and chronic NMN treatment in brain-specific SIRT1 knockout (BKO) and littermate control mice.

## Materials and methods

### Animals

Nestin-Cre hemizygous transgenic mice (Nes-Cre/+; B6.Cg-Tg(Nes-Cre)1Kln/J purchased from The Jackson Laboratory, #003771) were crossed with homozygous *loxP*-flanked exon 4 *Sirt1* allele mice (Sirt1^flox/flox^; B6.129-*Sirt1*^*tm3Fwa*^/DsinJ purchased from The Jackson Laboratory, #029603)^18^. Nes-Cre/+; Sirt1^flox/+^ were then crossed to Sirt1^flox/flox^ mice to generate Sirt1^flox/flox^ (WT) and Nes-Cre/+; Sirt1^flox/flox^ (BKO; brain-specific SIRT1 KO). Males were used for all studies to exclude the influence of hormonal fluctuations over the estrous cycles. All mice were maintained in the C57BL/6 background and were individually housed on a 12 h light/dark (LD) cycle with room temperature at 23 ± 1 °C. Food and water were available ad libitum.

All experimental procedures were approved by the Stanford University Institutional Animal Care & Use Committee (#21646) and were conducted in accordance with institutional ethical regulations and the ARRIVE guidelines (https://arriveguidelines.org). All efforts were made to minimize animal suffering or discomfort and to reduce the number of animals used. Euthanasia was performed in accordance with the AVMA Guidelines for the Euthanasia of Animals.

### Telemetry implantation and EEG/EMG surgery

Male mice were anesthetized with 3% isoflurane and implanted telemetry and EEG/EMG electrodes for sleep and circadian studies. A telemetry device (G2 E-Mitter; Mini Mitter OR, USA) was implanted in the abdominal cavity under 3% isoflurane anesthesia. A headstage with 4 EEG and 2 EMG electrodes were surgically implanted on the skull where two EEG electrodes were screwed over the motor cortex (1.0 mm lateral and 1.0 mm anterior to bregma) and the other two electrodes over the visual cortex (3 mm lateral and 1 mm anterior to lambda). Two Teflon-coated stainless-steel wires for EMG were placed into the neck extensor muscles. Mice were singly housed and allowed to recover from surgery for 2 weeks.

### Free-running experiments

Radiotelemetry-implanted mice were habituated to the recording condition for at least 1 week under 12:12 LD conditions (lights on 7 am, off 7 pm). The mice were released into constant dark (DD) conditions. For 3 weeks thereafter, mice (8 for each genotype) were maintained with regular chow under DD and then fed with high fat diet (60% kcal from fat, Research Diets, D12492) under DD for 3 more weeks. Spontaneous activity data were double-plotted in actograms using 1 min bin. χ^2^ periodogram analysis was performed using an in-house software to calculate circadian rhythm period length (tau) of locomotor activity for each mouse^19^.

### Sleep recording and analyses

Mice were tethered to recording cables that connect to a low-torque slip-ring commutator (Biella Engineering, Irvine, CA, USA). After 1 week of habituation to the cable and recording environment, consecutive recordings of EEG/EMG, locomotor activity and core body temperature were started. Following the baseline recording day, sleep deprivation was performed with gentle handling for 6 h after the light phase begins (Zeitgeber time: ZT 0–6). To avoid additional stress, we minimized touching the mice directly. Mice were allowed to sleep ad libitum without any disturbances after ZT6. EEG and EMG signals were acquired using a Grass amplifier (West Warwick, RI, USA), filtered (30 Hz Low Pass Filter for EEG; 10–100 Hz Band Pass Filter for EMG) and captured at a sampling rate of 256 Hz using a sleep recording system (Vital Recorder; Kissei Comtec Co. Ltd., Matsumoto, Japan). Locomotor activity was monitored through VitalView software (STARR Life Sciences Co., PA, USA) and analyzed in 1-hour bins.

EEG and EMG signals were automatically scored in 10 s epochs using SleepSign software (Kissei Comtec Co. Ltd., Matsumoto, Japan). According to standard criteria, each epoch was classified as Wake, NREM, or REM sleep. The automatic scoring output was visually examined and confirmed by experienced scorers, blind to genotype and treatment. Fast Fourier transform (FFT) was performed to analyze power spectra profiles of EEG over a 0–30 Hz with 0.5 Hz bins for the δ (0.5–4 Hz) bandwidth in a state-specific manner. Epochs with artifacts and noise were excluded from the calculation.

### Short- and long-term NMN administration

NMN powder was obtained from Jiangsu Chengxin Pharmaceutical Co., Ltd. (Jiangsu, China) and stored at −20 °C. For short-term administration, NMN solution was prepared in sterile saline and was administered intraperitoneally at the dose of 500 mg/kg body weight per day for consecutive 10 days. The dose regimen was determined by the previous report^11^. Each injection was performed between ZT2 and ZT5. Data recording was started from the next day after the last injection of vehicle or NMN. Locomotor activity and sleep data were analyzed and averaged for the following 2 days (day 11 and 12). For long-term administration, the dose regimen was determined based on the previous report^12^. Water consumption was measured for 2 weeks prior to the start of the chronic administration. NMN was then administered for 2 months in drinking water ad libitum at 300 mg/kg/day, based on the previously measured water consumption. The NMN solution was prepared weekly by dissolving NMN into autoclaved water at the respective doses and filtering for sterility. Water bottles and cages were changed weekly. Locomotor activity and sleep data for 2 days in the 9th week were analyzed and averaged.

### Quantitative real-time RT-PCR

After the completion of sleep recording, mice (< 50 g of body weight) were deeply anesthetized with isoflurane before cervical dislocation and decapitation. The whole brain and liver were quickly removed. Coronal sections at a thickness of 3 mm, 0.0 to −3.0 mm from bregma, were sliced on ice using a mouse brain matrix (Ted Pella). The sections were cut half in the middle, and each hemisphere and liver were snap-frozen in dry ice. Total RNA was extracted from one hemisphere using TRIzol (Invitrogen) and reverse-transcribed into cDNA with the SuperScript III reverse transcriptase (Invitrogen). Quantitative real-time RT-PCR was performed with the TaqMan Fast advanced PCR Master mix and TaqMan primers for each gene in the ViiA7 Real-Time PCR system (Applied Biosystems). The mRNA expression values were normalized to the level of *Gapdh* and were calculated using the delta-delta CT method. The following TaqMan probes were used: *Sirt1* (Mm00490758_m1), *Sirt2* (Mm01149204_m1), *Sirt3* (Mm00452131_m1), *Sirt4* (Mm01201915_m1), *Sirt5* (Mm00663723_m1), *Sirt6* (Mm01149042_m1), *Sirt7* (Mm01248607_m1), and *Gapdh* (Mm99999915_g1).

### Western blot

The other hemisphere and liver were homogenized in ice-cold RIPA lysis buffer. The homogenate was centrifuged at 14,000 g for 15 min and the supernatant was collected for western blotting. The protein concentration was determined using a BCA protein assay kit (Thermo Scientific). Samples were separated with 10% SDS/PAGE gels. Proteins were transferred onto PVDF membranes and probed with primary antibodies against SIRT1 (1:1000, 17–131, Millipore) and GAPDH (1:10000, ABS16, Millipore) followed by incubation with corresponding horseradish peroxidase-conjugated secondary antibodies. Blots were detected with SuperSignal West Dura (Thermo Scientific) and imaged using FluorChem E (ProteinSimple). We quantified the images using ImageJ (National Institute of Health).

### Statistical analyses

Statistical analysis was performed using GraphPad Prism 9 (GraphPad Software Inc.). The data from RT-PCR were analyzed by multiple unpaired t-test followed by Holm-Sidak’s method. The protein expression was analyzed using unpaired t-test. The sleep/wake parameters and time course changes in locomotor activity and FFT power spectrum were analyzed using two-way repeated measures ANOVA. For multiple comparisons, the Sidak correction was used. The level of statistical significance was set at *p* < 0.05. All data are shown as mean ± SEM.

## Results

### Brain-specific knockout of *Sirt1* and circadian phenotypes in SIRT1 BKO mice

**Fig. 1:**
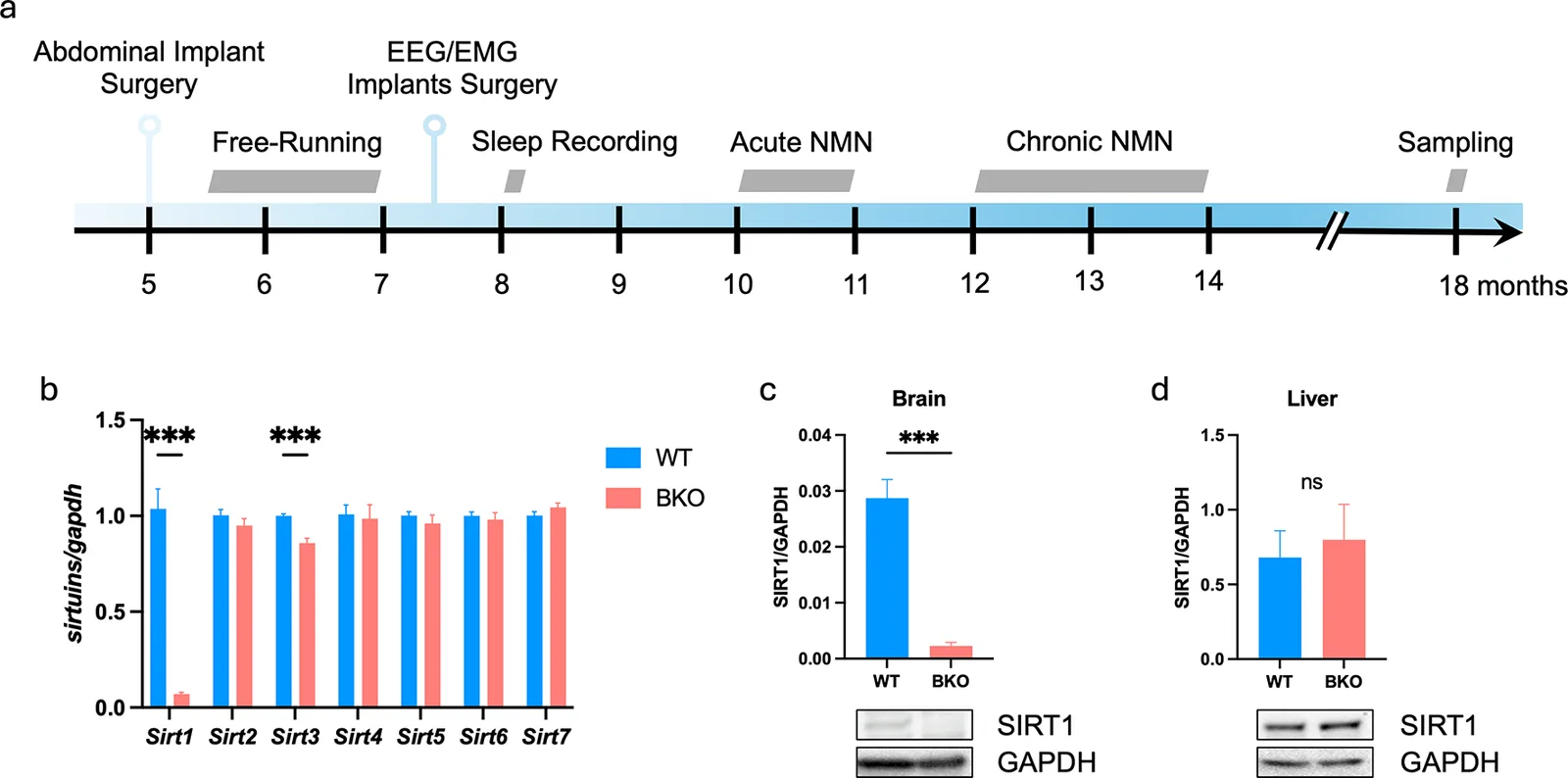
**Brain-specific knockout of** ***Sirt1*** **gen e and its protein in BKO mice.** (a) Schematic diagram of the experimental design. (b) Comparison of mRNA expression of 7 sirtuins in the brain (*n* = 7 for each genotype). (c) SIRT1 expression in the brain and liver (*n* = 7 for each genotype). Data is presented as mean ± SEM. ***, *P* < 0.001.

In this study, we characterized sleep and circadian phenotypes of SIRT1 BKO mice under basal condition and 6-hour sleep deprivation and further evaluated the effect of short- and long-term NMN treatment on sleep (Fig. 1a). To validate *Sirt1* gene knockout in the brain, we quantified the expression level of seven sirtuin genes, sirtuins 1–7. BKO mice completely knocked out the *Sirt1* expression, while other sirtuins, except *Sirt3*, were comparable between genotypes (Fig. 1b). *Sirt3* expression was slightly but significantly reduced in BKO mice. The SIRT1 protein expression was abolished in the brain, but not the liver of BKO mice (Fig. 1c, d). Thus, we confirmed brain-specific Sirt1 knockout at both mRNA and protein levels in BKO mice that were used for sleep and circadian experiments.

**Fig. 2:**
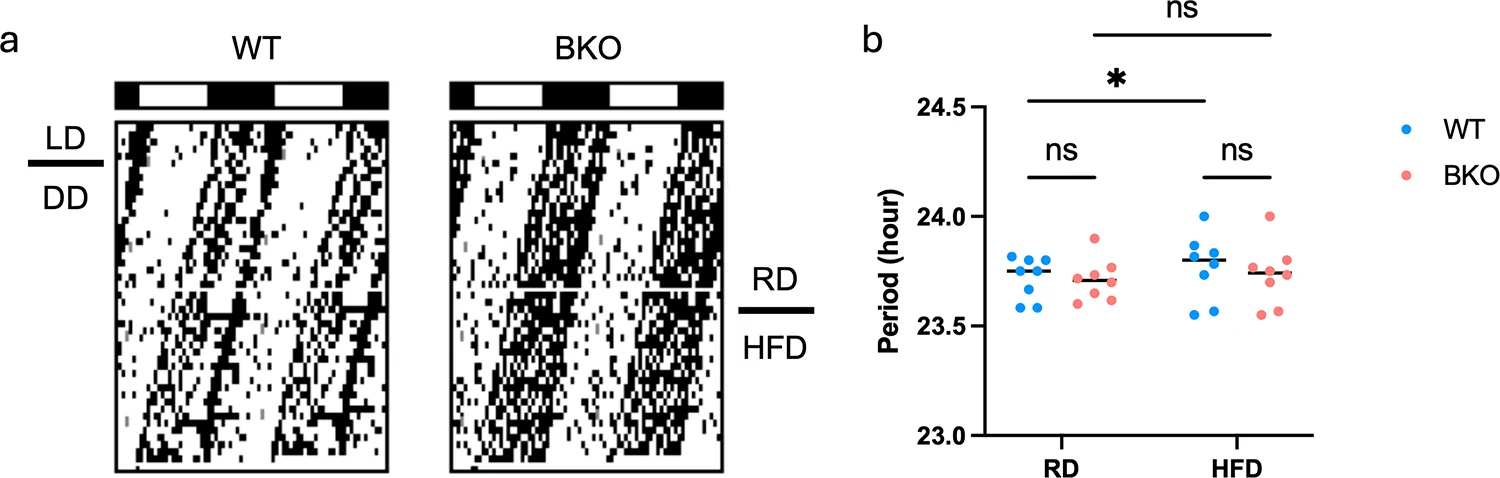
Circadian phenotype of BKO mice. (a) Representative actograms for WT and BKO mice are shown. Circadian period for each mouse was calculated by periodogram (*n* = 8 per genotypes). Actograms are double-plotted, showing 2 days per row. LD, 12 h light/dark; DD, constant dark; RD, regular diet; HFD, high fat diet. Data is presented as mean ± SEM. *, *P* < 0.05.

The previous circadian study in SIRT1 BKO mice led us to examine the circadian rhythm in our setting^20^. We used radiotelemetry implants to monitor locomotor activity continuously and calculate the circadian period^19^. However, unlike the previous report, there was no difference in the circadian period between BKO and littermate WT mice (WT vs. BKO, 23.72 ± 0.03 vs. 23.71 ± 0.03, *p* = 0.89) (Fig. 2). High fat diet is known to lengthen the circadian period^22^ and it is of interest to test the stress responses impacting both metabolism and circadian rhythm in BKO mice. High fat feeding slightly altered the period of the locomotor activity in WT mice. The influence of high fat was likely mitigated in BKO mice, although the impact on the period length was limited (regular diet vs. high fat diet: 23.72 ± 0.03 vs. 23.77 ± 0.05, *p* = 0.03, for WT; 23.71 ± 0.03 vs. 23.73 ± 0.05, *p* = 0.30, for BKO).

### Sleep phenotypes of SIRT1 BKO mice

It has been reported that whole-body KO or brain-specific overexpression of SIRT1 alters sleep-wake profiles and EEG spectral power^9,10^. SIRT1 regulates energy metabolism in peripheral tissues and systemic deletion of *Sirt1* can indirectly interfere with brain functions. To circumvent this issue and evaluate the role of brain SIRT1, we analyzed baseline sleep and the responses to sleep deprivation in BKO mice.

**Fig. 3:**
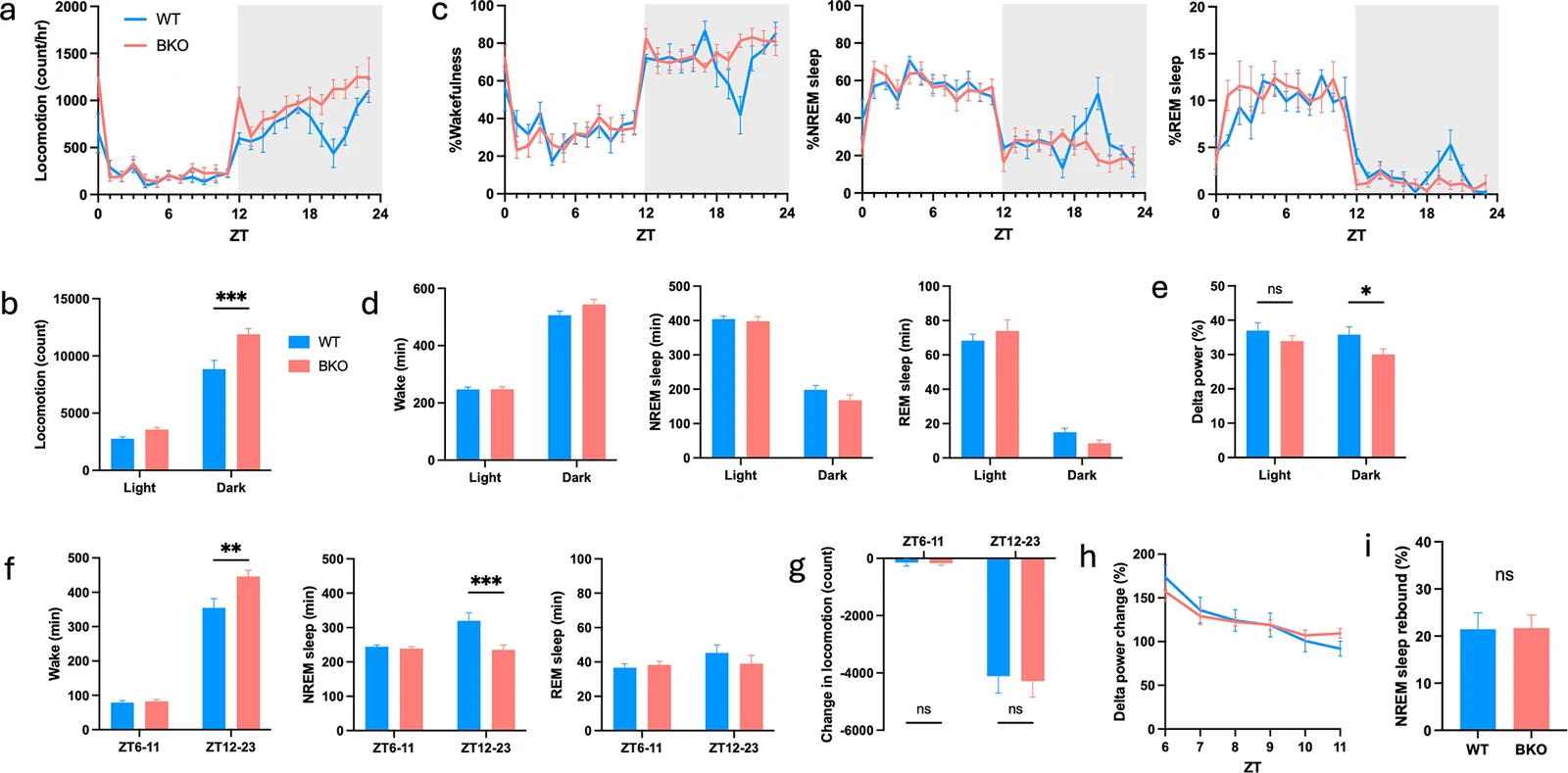
Sleep phenotype under basal and sleep-deprived conditions in BKO mice. a-e, Analysis of spontaneous activity (a, b), sleep/wake amounts (c, d), and delta power during NREM sleep (e, WT vs. BKO in light and dark periods) in baseline (*n* = 8 for each genotype). f-i, Analysis of sleep/wake amounts (f) after 6-hour sleep deprivation (ZT0-5), and the change in spontaneous activity (g), delta power (h), and rebound sleep (i) during recovery sleep (ZT6-11). Shaded areas represent the dark period. Data is presented as mean ± SEM. ZT, zeitgeber time. *, *P* < 0.05; **, *P* < 0.01; ***, *P* < 0.001.

In the baseline day, BKO mice had a higher spontaneous activity than WT mice during active period (Fig. 3a, b). However, there were no differences in sleep architecture and amount of each vigilance state between genotypes (Fig. 3c, d). FFT analysis showed that BKO mice had a lower delta power during NREM sleep in the dark period (WT vs. BKO: *p* = 0.0447) (Fig. 3e).

The interplay of two primary factors, circadian rhythms and sleep homeostasis, is fundamental to sleep regulation. We performed sleep deprivation to evaluate sleep homeostasis. Sleep deprivation in the first 6 h of the light period induced altered responses in sleep and wake behaviors during the following dark period, but not recovery period (ZT6-11), in BKO mice. In contrast to the baseline condition, BKO mice spent more time in wake and less time in NREM sleep during the dark period than WT mice (Fig. 3f, Suppl Fig. 1). While BKO mice showed a high spontaneous activity during the dark period, the impact of sleep deprivation was comparable between genotypes (Fig. 3g, Suppl Fig. 1). Changes in delta power and NREM sleep rebound were calculated by comparing each parameter during the period from ZT6-11 between baseline and sleep deprivation. There were no differences in the increase of delta power and NREM sleep rebound between genotypes (Fig. 3h, i), indicating that sleep homeostasis remains largely intact in BKO mice.

### Short-term intraperitoneal administration of NMN

**Fig. 4:**
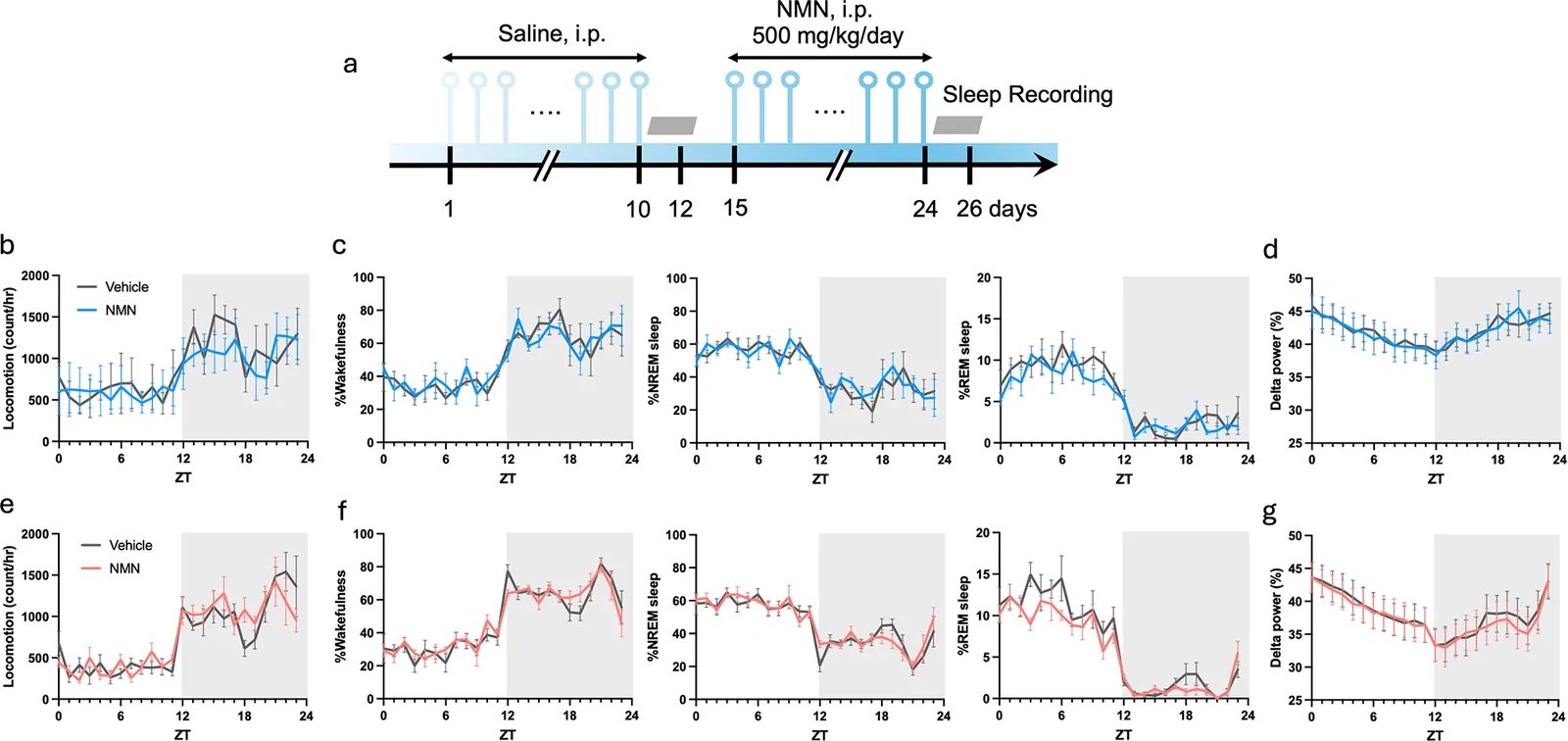
Effects of short-term NMN administration on sleep. (a) Schematic diagram of the experimental design of acute NMN treatment. Sleep data was recorded for 2 days (Days 11–12 for vehicle, 25–26 for NMN). b-d, Comparison of spontaneous activity (b), sleep/wake amounts (c), and delta power during NREM sleep (d) in WT mice (*n* = 6). e-g, Comparison of spontaneous activity (e), sleep/wake amounts (f), and delta power (g) in BKO mice (*n* = 6). Four mice (2 for each genotype) were excluded from data analysis due to high EEG/EMG noises. Shaded areas represent the dark period. Data is presented as mean ± SEM. ZT, zeitgeber time.

We hypothesized that NMN might help regulate the sleep-wake cycle and locomotor activity, potentially through its role in NAD+ production, and that those effects could be blunted in BKO mice. In the same group of mice at 10 months of age, sterile saline or 500 mg/kg of NMN were repeatedly given intraperitoneally for consecutive 10 days (Fig. 4a). The same dose regimen was used in the previous study in which NMN improves glucose intolerance and lipid profiles in age-induced type 2 diabetes mice^11^. Despite its beneficial effects in the peripheral metabolic organs, the acute NMN treatment had no influence on all aspects of sleep and wake behavior (i.e., locomotion, sleep architecture, and delta power) in WT mice (Fig. 4b-d). Similarly, BKO mice had no alteration in sleep and wake behavior with NMN treatment (Fig. 4e-g).

### Long-term oral administration of NMN

**Fig. 5:**
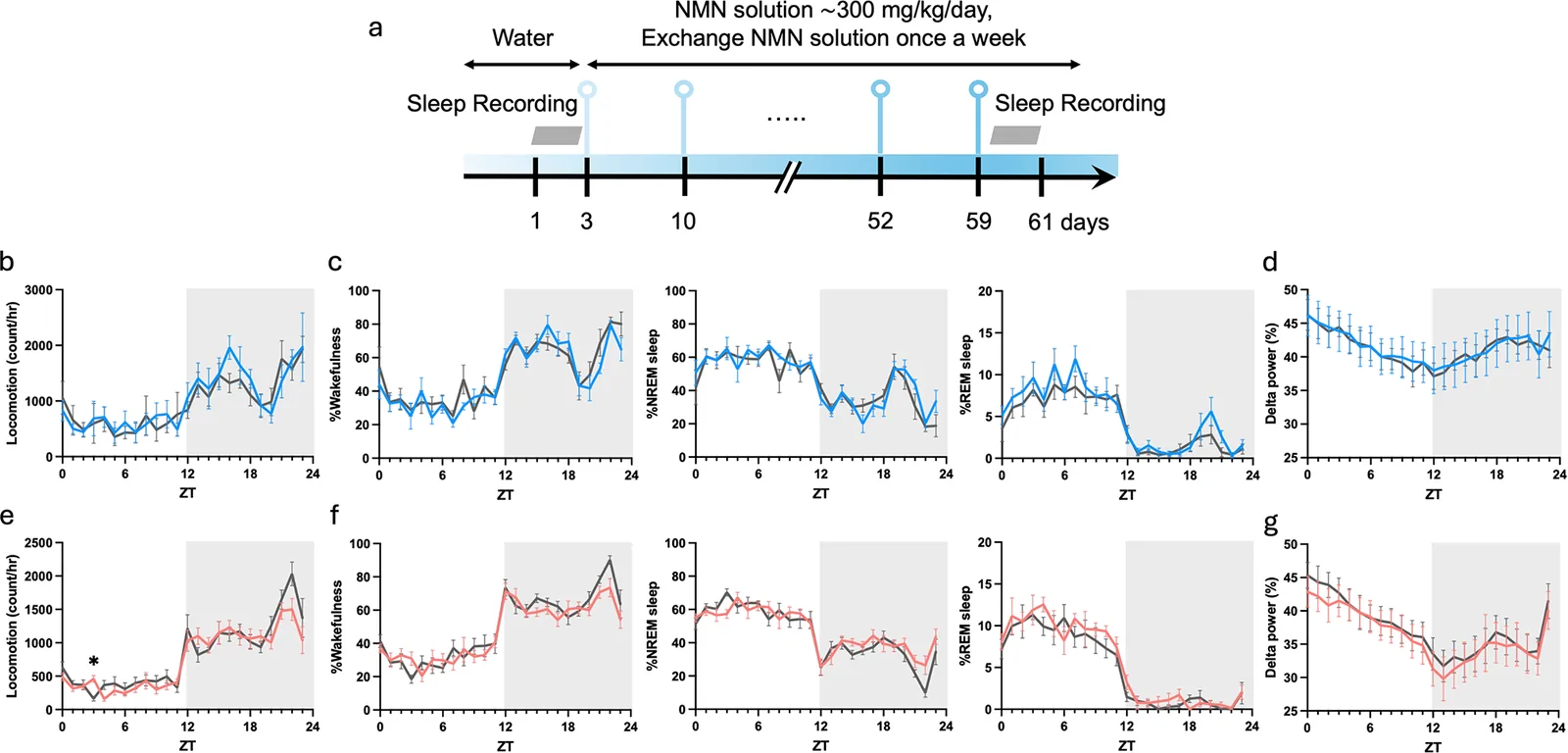
Effects of long-term NMN administration on sleep. (a) Schematic diagram of the experimental design of chronic NMN treatment. Sleep data was recorded for 2 days under NMN treatment (Days 1–2 for regular water, 60–61 for NMN). b-d, Comparison of spontaneous activity (b), sleep/wake amounts (c), and delta power during NREM sleep (d) in WT mice (*n* = 6). e-g, Comparison of spontaneous activity (e), sleep/wake amounts (f), and delta power (g) in BKO mice (*n* = 6). Four mice (2 for each genotype) were excluded from data analysis due to high EEG/EMG noises. Shaded areas represent the dark period. Data is presented as mean ± SEM. ZT, zeitgeber time. *, *P* < 0.05.

Since long dosing regimens are likely more realistic and might help greater benefit of NMN than the short-course regimen, we then conducted a 2-month-long NMN administration in drinking water (Fig. 5a). The dose regimen was determined based on the previous study in which a 12-month-long NMN administration effectively mitigates age-associated physiological decline in mice^12^. We have not observed any behavioral adverse effects over the course of treatment. However, NMN had no influence on all aspects of sleep and wake behavior in neither WT nor BKO mice, even in the chronic treatment (Fig. 5b-g). Our data clearly show that NMN has no direct effect on sleep and wake behavior in middle-aged mice.

## Discussion

In this study, our results provide strong evidence that brain SIRT1 functionally plays minor roles in the regulation of sleep and wake behavior. Unlike whole-body KO mice, the specific loss of brain SIRT1 did not alter the amount of wake, NREM, and REM sleep, and both circadian rhythm and sleep homeostasis were also unaffected. It appears that severe wake impairments and morphological changes to wake-active neuron in outbred KO mice would potentially be caused by developmental defects^18,22^. On the other hand, the current study supports the previous finding of alterations in delta power during NREM sleep: blunted delta power in outbred KO mice and the enhancement in overexpressing mice. The mechanism is unknown, but a subset of neurons in the dorsomedial hypothalamus has recently been identified as a key subpopulation that is responsible for the aging process controlled by SIRT1^23^. Interestingly, the subset of cells sends projections to some areas regulating sleep/wake states, such as the preoptic area, the paraventricular nucleus, the lateral hypothalamus, and the periaqueductal gray. We speculate that lower sleep drive in baseline sleep and enhanced wakefulness after sleep deprivation may be partly involved in disturbed sleep in challenging physiological and pathological conditions that lower SIRT1 activity in the brain.

While many clinical trials involving NMN have been carried out, this is the first preclinical study testing sleep. Five clinical trials have evaluated subjective sleep quality in middle-aged or older adults. These studies have demonstrated the potential of NMN to improve physical performance but also have shown mixed results of the efficacy on sleep quality: no sleep improvement^24,25^ and better sleep quality and less drowsiness^15–17^. In the current study, neither acute nor chronic NMN treatment changed sleep duration and quality in middle-aged WT mice. Considering our findings, preclinical studies on peripheral tissues, and clinical observation, a possible explanation is that the improvement in muscle function and energy metabolism may indirectly contribute to the potential benefits of NMN on sleep quality and fatigue in the elderly^11–15,26,27^.

Prior work showed that brain-specific loss or overexpression of SIRT1 led to altered central circadian rhythm by modulating circadian gene expression^20^. However, contrary to the work, we found no changes in intrinsic circadian periods between WT and BKO mice. Monitoring daily activity rhythms using running wheels or telemetry system is the commonly used and well-established method for the assessment of circadian phase. Wheel running is viewed as a reinforcing behavior rather than a fundamental locomotor activity in mice. It enhances adaptability to artificial jet lag and high fat feeding and influences peripheral rhythms in the liver and muscle^28–30^. Our telemetry system monitors behavioral rhythms without altering locomotor rhythm^19^. However, despite some differences in experimental variables (e.g., methods in data acquisition and analysis), to our knowledge, the reason for these inconsistent results is unknown, which is a potential limitation of the study. Thus, the role in activity rhythm remains inconclusive and warrants further investigation. Regarding circadian regulation, the mechanisms responsible for the circadian clock control have been the subject of contradictory findings: one mechanism proposes *Sirt1* as a positive regulator of circadian components through PER2 deacetylation and its degradation^5^, while the other mechanism proposes it as a negative regulator through deacetylation of BMAL1 and histone H3^6^. Our results may reconcile these seemingly contradictory conclusions. In addition, we found that the response to high fat diet was slightly attenuated in terms of circadian periods in BKO mice. Whereas the expression or rhythmicity of core clock genes in the hypothalamus are less affected by high fat feeding, those of neuropeptides, including orexin/hypocretin, are more affected^22^. For example, high fat diet dampens diurnal rhythmicity in orexin gene expression. Interestingly, *Sirt1*-mediated orexin type 2 receptor (*Ox2r*) expression in the hypothalamus is critical for longevity-associated phenotypes^20^. BKO mice might be tolerant to high fat diet due to perturbed *Sirt1*/*Ox2r* signaling. Further studies are warranted.

There are two limitations in the NMN supplementation study. First, we administered NMN solution to middle-aged mice ranging from 10 to 14 months old. There are two reasons that the ages were chosen in the current study: first, no data about lifespan available in BKO mice; and second the deterioration of EEG/EMG signals over time. In general, aged mice would be ideal to see the anti-aging effects of the NMN replenishment because NAD+ declines with age^31^. Importantly, it has been reported in the mouse brain that NAD+ levels gradually decrease with age, reaching 63% in 10- to 12-month-old mice compared to that of 1-month-old mice^32^. Thus, NMN replenishment would be sufficient at these ages. Second, related to the first limitation, long-term administration was performed for only 2 months due to the same reason.

Disruptions of circadian rhythms and sleep cycles are frequently observed in aging and neurodegenerative diseases such as Alzheimer’s disease. These disruptions can be a potential contributor to the development of disease and its progression, indicating that they would be a promising therapeutic target. Overall, this study presents the controversial role of *Sirt1* in sleep and circadian rhythm and provides robust preclinical evidence for NMN to better understand its efficacy.

## Supplementary Information

Below is the link to the electronic supplementary material.

Supplementary Material 1

Supplementary Material 2

## Data Availability

The datasets used and/or analyzed in the current study are available from the corresponding author on reasonable request.
